# The Role of CAM in Public Health, Disease Prevention, and Health Promotion

**DOI:** 10.1155/2015/528487

**Published:** 2015-12-24

**Authors:** Cheryl Hawk, Jon Adams, Jan Hartvigsen

**Affiliations:** ^1^Northwest Center for Lifestyle and Functional Medicine, University of Western States, Portland, OR 97230, USA; ^2^Australian Research Centre in Complementary and Integrative Medicine (ARCCIM), University of Technology Sydney, Sydney, NSW 2007, Australia; ^3^Department of Sports Science and Clinical Biomechanics, Nordic Institute of Chiropractic and Clinical Biomechanics, University of Southern Denmark, 5230 Odense M, Denmark

As the worldwide burden of chronic disease continues to rise, disease prevention and health promotion become increasingly important components of public health. Although many complementary and alternative medicine (CAM) practices have emphasized health promotion and the area of CAM health care holds much opportunity and challenge for issues of public health, CAM research to date has been dominated by more clinically restrictive issues [1, 2]. However, CAM practitioners can constitute a public health resource to increase the population's access to certain clinical preventive services if integrated into mainstream public health practice. As part of this integration of CAM practices, it is important to investigate methods of effective interprofessional collaboration involving both CAM and mainstream professions. It is also essential to identify issues that might be challenging to integration from the perspective of both mainstream and CAM providers such as professional biases on both sides, differences in perception of health and disease, common language, and adherence to evidence-based principles.

This special issue includes original research papers and systematic reviews investigating the issues related to CAM practices in the public health arena as well as outcomes of CAM treatment for chronic disease prevention and/or management (tertiary prevention).

There are 14 articles in this issue, from six countries and on a wide variety of topics. The topics addressed are almost equally divided between treatment of specific conditions of public health importance and disease prevention through risk factor reduction. A smaller number of articles address issues related to traditional medicine use among the general public. Articles on treatment of conditions include a large clinical study by J.-F. Tang et al. on safety and evaluation of Danhong injection; a basic science study on* Brucea javanica* oil by Y. Liu et al.; a systematic review and meta-analysis of the efficacy of compound kushen on cancer pain by X. Ma et al.; an analysis of Qigong's effect on chronic fatigue syndrome by L. P. Yuen et al.; a systematic review of acupuncture for allergic rhinitis and asthma by L. Zhou et al.; and an analysis of the effects of guided imagery on patients on mechanical ventilation by B. Brakovich et al.

Articles on prevention and risk factor reduction were a randomized controlled trial of Tai Chi on weight loss, metabolic syndrome, and bone mineral density by T. C.-Y. Kwok et al.; an analysis of body constitution through Traditional Chinese Medicine by C.-H. Lee et al.; a survey of apitherapy use for disease prevention by S. Trumbeckaite et al.; an exploration of the possible effect of CAM on antibiotic resistance by W. B. V. Leeuwen et al.; and a survey of the effect of Traditional Chinese Medicine on decreasing stroke risk among users of corticosteroids for dermatitis by J.-L. Shen et al.

Finally, F. Xu et al. described the validation of a questionnaire based on Traditional Chinese Medicine to assess health status; S. H. Ng et al. described use of the Theory of Planned Behaviour related to Traditional Chinese Medicine use; and G. D. Hughes et al. characterized the use of herbal medicines for noncommunicable disease.

These articles tended to focus more on single herbal or other traditional preparations or procedures for both treatment and prevention, with very little emphasis on health behaviour, which is typically a cornerstone of public health programs. Behaviours related to diet, physical activity, and stress reduction are known to be increasingly important factors in determining the health of the public and are also areas of importance and emphasis for many CAM practitioners. It is necessary for CAM professions to become visible in this arena and for CAM research to extend into this direction rather than focusing narrowly on specific therapies if these efforts are to be recognized and utilized for the benefit of populations.

*Cheryl Hawk* *Cheryl Hawk* *Jon Adams* *Jon Adams* *Jan Hartvigsen* *Jan Hartvigsen*
